# Novel mouse mammary cell lines for *in vivo* bioluminescence imaging (BLI) of bone metastasis

**DOI:** 10.1186/1480-9222-14-6

**Published:** 2012-04-17

**Authors:** Celeste Bolin, Caleb Sutherland, Ken Tawara, Jim Moselhy, Cheryl L Jorcyk

**Affiliations:** 1 Department of Biological Sciences, Boise State University, Boise, ID, USA; 2 Present Address: Department of Cancer Biology, University of Arizona, Tucson, AZ, USA

**Keywords:** Breast cancer, Mammary cancer, Bone metastasis, *in vivo* imaging, 4 T1 cells, 4 T1.2 cells, Osteolysis, Syngeneic Balb/c model

## Abstract

**Background:**

Tumor cell lines that can be tracked *in vivo* during tumorigenesis and metastasis provide vital tools for studying the specific cellular mechanisms that mediate these processes as well as investigating therapeutic targets to inhibit them. The goal of this study was to engineer imageable mouse mammary tumor cell lines with discrete propensities to metastasize to bone *in vivo*. Two novel luciferase expressing cell lines were developed and characterized for use in the study of breast cancer metastasis to bone in a syngeneic mouse model.

**Results:**

The 4 T1.2 luc3 and 66c14 luc2 cell lines were shown to have high levels of bioluminescence intensity *in vitro* and *in vivo* after orthotopic injection into mouse mammary fat pads. The 4 T1.2 luc3 cell line was found to closely model the sites of metastases seen in human patients including lung, liver, and bone. Specifically, 4 T1.2 luc3 cells demonstrated a high incidence of metastasis to spine, with an *ex-vivo* BLI intensity three orders of magnitude above the commercially available 4 T1 luc2 cells. 66c14 luc2 cells also demonstrated metastasis to spine, which was lower than that of 4 T1.2 luc3 cells but higher than 4 T1 luc2 cells, in addition to previously unreported metastases in the liver. High osteolytic activity of the 4 T1.2 luc3 cells *in vivo* in the bone microenvironment was also detected.

**Conclusions:**

The engineered 4 T1.2 luc3 and 66c14 luc2 cell lines described in this study are valuable tools for studying the cellular events moderating the metastasis of breast tumor cells to bone.

## Background

Metastasis of breast carcinoma cells from the primary tumor to secondary organ sites such as lung, liver, brain, and bone is the leading cause of mortality in patients with breast cancer [1]. Bone is the most frequent site of metastasis in breast cancer patients, observed in approximately 65 to 75% of patients with metastatic breast cancer [2]. Bone metastases often cause significant pain and morbidity in these patients due to osteolysis and bone resorption, and the median survival time after detection these metastases is approximately two years [3,4].

Researchers studying breast tumorigenesis and metastatic progression *in vivo* utilize several types of mouse models including transgenic, xenograft, and syngeneic mouse models. Transgenic mouse models that generate spontaneous mammary tumors have been developed using promoters such as the mouse mammary tumor virus (MMTV) promoter to drive oncogenes, including polyoma middle T antigen (MMTV-PyMT) and ErbB2/Neu (MMTV-Neu) (for review see [5]). Transgenic mice lacking the p53 tumor suppressor gene (p53 ^−/−^), which is mutated in 40-50% of human breast cancers, have also been utilized extensively in cancer studies but do not reproducibly form mammary tumors [6]. A major drawback to these transgenic models, along with the commonly used C3(1)-SV40 T-antigen transgenic mice that also develop mammary tumors independent of steroid supplementation, is that bone metastases cannot be detected (for review see [7]). Given that these tumors occur spontaneously via transformation of the host’s normal cells that do not have specific, imageable cellular tags, it is very difficult to track these cells *in vivo* during tumor progression and metastasis.

In the xenograft mouse model, human breast cancer cells are injected into immunocompromised, athymic mice. This model is useful because it allows breast cancer cells of human origin to be studied. However, when studying the metastatic cascade in these models, in which the host organism and the implanted tumor cells are not of the same species, important tumor and host stromal interactions may be disrupted due to interspecies signaling incompatibilities [8]. Additionally, due to the compromised immune system of the athymic mouse, immune-mediated tumor and host stromal interactions important for metastasis may be lost in this model (for review see [9]).

Syngeneic models in which the tumor cells are placed into the same species they originated from, and therefore the tumor microenvironment is of the same species, are able to overcome limitations of both xenograft and transgenic models in studying metastasis. Commonly used syngeneic mouse models of breast cancer utilize cells isolated from mammary tumors that occurred spontaneously in wild-type Balb/c mice. These cells can then be injected orthtopically into Balb/c mice and will reproducibly grow a mammary tumor. Currently, there is a series of mouse mammary cancer cell lines derived from the spontaneous breast tumors of Balb/c mice including non-metastatic 67NR cells, 66c14 cells that metastasize to lung, and 4 T1 cells that metastasize to lung and liver [10]. 4 T1.2 cells are a sub-clone of the original osteolytic 4 T1 mammary tumor cell line that have been selected for their increased propensity to metastasize to bone [10]. While 66c14 cell metastasis is restricted to the lymph nodes and lung, 4 T1.2 cells closely resemble the metastatic profile in humans with metastasis to the bone, lymph nodes, and lung [10,11]. Additionally, the 4 T1.2 orthotopic model results in an increase in hypercalcemia due to osteolysis, an important characteristic that resembles human bone metastasis [12]. Engineering these mouse cells to be traceable and imageable in a live animal make them even more valuable for studying metastasis to bone *in vivo* in a syngeneic mouse model.

Currently, there are several human breast cancer cell lines engineered to express bioluminescence imaging (BLI) tags such as luciferase (luc) that can be used to track the metastases of these cells *in vivo*. These cell lines include MDA-MB-231-luc2 and MCF-7-luc2, both available commercially (Caliper Life Sciences, Hopkinton, MA). In general, human mammary cancer cells utilized in xenograft mouse models of breast cancer demonstrate a low propensity to metastasize to bone unless introduced via intra-cardiac injection, which bypasses critical initial steps in the metastatic cascade (for review see [13]). To date, there are only two mouse mammary cancer cell lines commercially available that stably express the luc gene, 4 T1-luc2 and 4 T1-luc2-GFP cells, available from Caliper Life Sciences (Hopkinton, MA, USA).

In this study we generated by transfection of 66c14 and 4 T1.2 mouse mammary carcinoma cells with the pGL4 vector two new cell lines that stably express the luciferase gene. These two cell lines, referred to as 66c14 luc2 and 4 T1.2 luc3, produce high levels of bioluminescence *in vitro* and *in vivo*. Additionally, tumor progression *in vivo* was consistent with the parental 66c14 and 4 T1.2 cell lines. Importantly, the overall metastatic burden, quantified as the number and photon density of BLI areas, was higher in mice injected orthotopically with the 4 T1.2 luc3 cells as compared to both 66c14 luc2 cells and the commercially available 4 T1 luc2 cells (Caliper Life Sciences, Hopkinton, MA). *In vivo* and *ex vivo* imaging of metastasis showed that the 4 T1.2 luc3 cells had a statistically significant higher level of metastases to the spine and lung as compared to the 66c14 luc2 cells, which nonetheless showed detectable levels of spine and lung metastases. Therefore, the newly established 4 T1.2 luc3 and 66c14 luc2 cell lines are reliable tools for monitoring and quantifying breast cancer metastasis to bone and other organs as well as for studying the signaling pathways associated with these processes.

## Results

### Establishment and characterization of 66c14 and 4 T1.2 mouse mammary carcinoma cell lines that stably express luciferase

**Figure 1:**
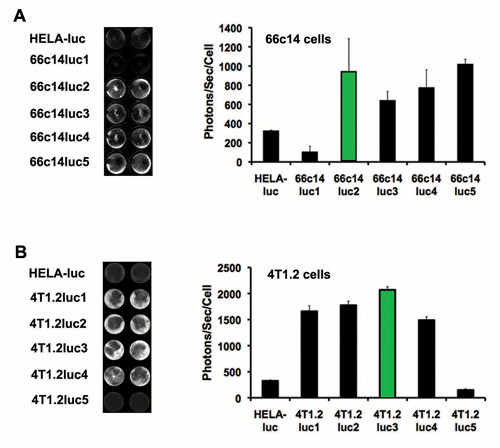
**Bioluminescence intensities of 66c14 luc- and 4 T1.2 luc-expressing cell lines *in vitro *.**Image analysis show bioluminescence from clonal populations of **(A)** 66c14 luc(1–5) cells and **(B)** 4 T1.2 luc(1–5) cells. Green bars highlight 4 T1.2 luc3 and 66c14 luc2 clones with high mean bioluminescence values. Data is expressed as photons/sec/cell (mean ± std dev, *n = 4 *) calculated by comparing each cell line to known values of HeLa-Luc cells (350 photons/sec/cell; see Materials and Methods).

Of the five different 66c14 luc lines produced, 66c14 luc5 cells produced the highest bioluminescence intensity, 1,019 photons/sec/cell, and the 66c14 luc2 cells produced the second highest, 945 photons/sec/cell (Figure 1A, green bar). Of the five different 4 T1.2 luc lines produced, 4 T1.2 luc3 cells produced the highest level of bioluminescence intensity, at 2,070 photons/sec/cell (Figure 1B, green bar). Two cell lines, 66c14 luc1 (105 photons/sec/cell) and 4 T1.2 luc5 (150 photons/sec/cell), had bioluminescence intensities below the minimum detection limit (MDL) for *in vivo* imaging (~300 photos/sec/cell; Caliper Life Sciences) and were not utilized in the following *in vivo* studies. The other eight luc-expressing cell lines generated in 4 T1.2 and 66c14 cells were further assessed for *in vivo* BLI based on *in vitro* bioluminescent intensities at least twice the *in vivo* MDL.

### *In vivo* tumor growth of 66c14 luc2 and 4 T1.2 luc3 cells

**Figure 2:**
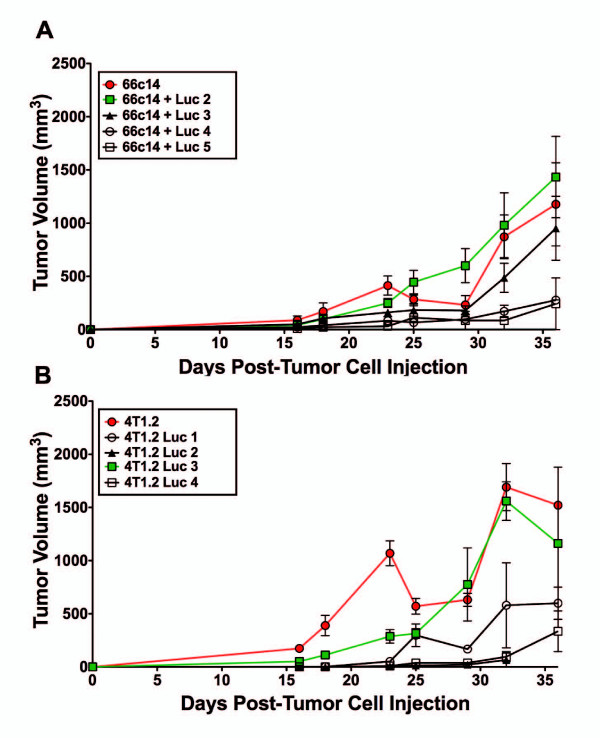
**Tumor growth of 66c14 luc and 4 T1.2 luc cells injected orthotopically into Balb/c mice.** 66c14 luc and 4 T1.2 luc cell lines were injected into the mammary fat pad of Balb/c mice and tumor volume was measured over 36 days. **(A)** 66c14 luc2 tumor growth (green/square) was most consistent with parental 66c14Tumor growth (red/circle) and **(B)** 4 T1.2 luc3 tumor growth (green/square) was most consistent with parental 4 T1.2 (red/circle) tumor growth. Data is expressed as tumor volume (mm^3^) (mean ± std dev; *n = 3*).

**Figure 3:**
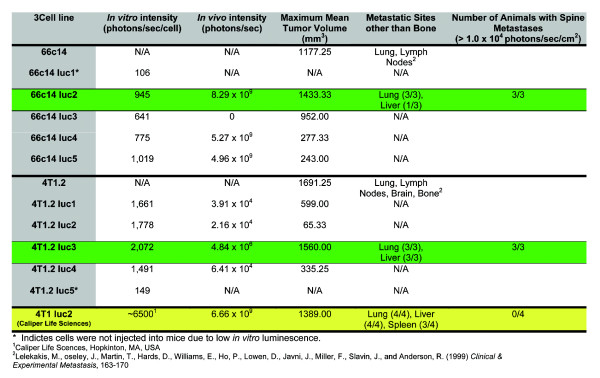
**Summary of *in vitro *and *in vivo *analysis of luc-expressing 4 T1.2 and 66c14 cell lines. **

To confirm that the luc-expressing cell lines grow *in vivo* in a manner comparable to the parental cells, tumor growth was monitored. 1 × 10^5^ cells (parental 66c14, 66c14 luc2, 66c14 luc3, 66c14 luc4, and 66c14 luc5 or parental 4 T1.2, 4 T1.2 luc1, 4 T1.2 luc2, 4 T1.2 luc3, and 4 T1.2 luc4) were injected into the mammary fat pad of female Balb/c mice (n = 3 per cell line) (Figure 2). Tumor size was measured using calipers twice a week for up to 36 days and expressed as tumor volume (mm^3^) = (length × width^2^/2) [11]. Mice injected with 66c14 luc2 cells (Figure 2A; green) displayed a tumor growth pattern similar to that of mice injected with parental 66c14 cells (Figure 2A; red). *In vivo* analysis of 66c14 luc3 cells demonstrated a small reduction in maximum tumor volume compared to parental 66c14 cells but did not produce bioluminescence *in vivo* (Figure 2A, Figure 3). Mice injected with 66c14 luc4 or luc5 cells (the cells with high BLI intensities *in vitro*) had severely reduced tumor growth *in vivo* (Figure 2A, Figure 3). Injection of 4 T1.2 luc3 cells (the cells with the highest BLI intensity *in vitro;* Figure 2B; green) resulted in a tumor growth pattern similar to that of parental 4 T1.2 cells (Figure 2B; red). 4 T1.2 luc1, luc2, and luc4 cell injections all resulted in reduced tumor progression *in vivo* (Figure 2B, Figure 3). Overall, 66c14 luc2 and 4 T1.2 luc3 cell lines demonstrated tumor progression most consistent with parental cell lines.

**Figure 4:**
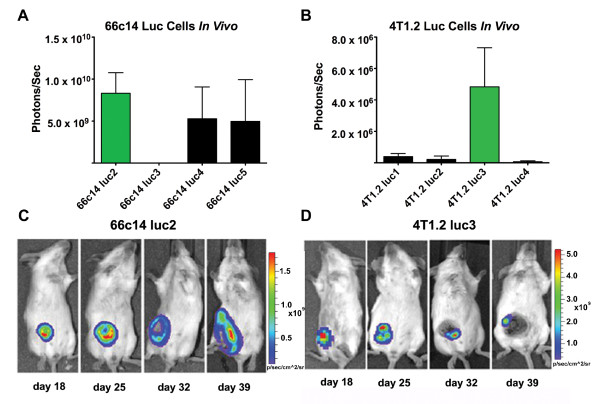
**BLI of 66c14 luc and 4T1.2 luc cells *in vivo *.** Tumor bioluminescence intensities from each of the **(A)** 66c14 luc and **(B)** 4 T1.2 luc tumor-bearing mice on day 25 after tumor cell injection are shown. 66c14 luc2 and 4 T1.2 luc3 exhibited the highest BLI intensity *in vivo *(green bars). Sequential images of **(C)** 66c14 luc2 and **(D)** 4 T1.2 luc3 tumor-bearing mice imaged ventrally by BLI once a week for 4 weeks are shown. 66c14 luc2 tumors continued to grow while 4 T1.2 luc3 tumors became necrotic between day 25 and 32. Data expressed as photon/sec (mean ± std dev; *n=3*).

Next, BLI intensity of 66c14 luc and 4 T1.2 luc cells during tumor growth was assessed *in vivo*. Tumors from injected 66c14 luc2 cells produced the highest level of BLI intensity of all the 66c14 luc cells, averaging 8.29 × 10^9^ photons/second on day 25 after tumor cell injection (Figure 4A; green, Figure 3). Tumors from injected 4 T1.2 luc3 cells produced the highest level of BLI intensity of all the 4 T1.2 luc cells, averaging 4.84 × 10^6^ photons/second on day 25 after tumor cell injection (Figure 4B; green, Figure 3). Sequential images on days 18, 25, 32, and 39 of mice injected with the 66c14 luc2 cell line showed increasing BLI intensity at the tumor site correlating with increasing tumor size (Figure 4C). The BLI intensity of tumors from mice injected with 4 T1.2 luc3 cells started to decrease after day 25, likely due to the tumor-associated necrosis (Figure 4D).

### BLI of metastases from mice injected with 66c14 luc2 or 4 T1.2 luc3 cells

The 66c14 luc2 and 4 T1.2 luc3 cell lines were chosen for additional investigation of metastatic potential due to their consistently similar tumor growth profiles with the parental cell lines, and high levels of BLI intensity *in vivo*. These two new cell lines were compared to the 4 T1 luc2 cell line purchased from Caliper Life Sciences described previously [14]. Independent orthotopic injection of each of these three cell lines (1 × 10^5^ cells) into the mammary fat pad of female Balb/c mice (n = 3) resulted in palpable tumors at day 14 after injection. Mice injected with the 66c14 luc2, 4 T1.2 luc3, or 4 T1 luc2 cells were imaged once the mice had reached endpoint criteria of maximum metastases including a loss of activity and 10% of their body weight (32 days after injection for the 66c14 luc2 and 4 T1.2 luc3 injected mice and 28 days for the 4 T1 luc2 injected mice).

**Figure 5:**
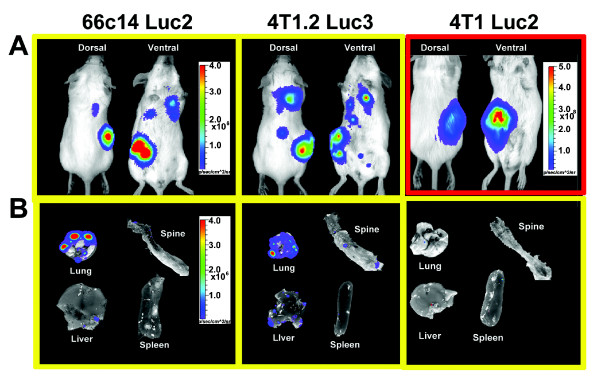
**Metastasis of 66c14 luc2, 4 T1.2 luc3, and 4 T1 luc2 cells *in vivo *.** Representative **(A)** whole animal and **(B)***ex vivo * organs from mice injected with the 66c14 luc2 (left), 4 T1.2 luc3 (middle), or commercially available 4 T1 luc2 (right) cells, 4–5 weeks after injection are shown. Primary tumor bioluminescence intensities were visualized in animals injected with all cell types in both dorsal and ventral views. Bioluminescence was also detectable in the thoracic region of the 66c14 luc2 and 4 T1.2 luc3 animals correlating with *ex vivo * bioluminescence from metastatic lesions detected in lung and spine. No bioluminescence representing metastatic lesions was detected in mice injected with 4 T1 luc2 cells. The bioluminescence intensity of the primary tumor from the 4 T1 luc2 mouse represented was very high and likely prohibited secondary sites of metastasis from being visualized. The BLI scale next to the whole body image of the 4 T1 luc2 injected mouse (highlighted in red) reflects this difference. All other images (highlighted in yellow) are presented with a consistent BLI scale. Overall, the 4 T1.2 luc3 cells showed the highest levels of BLI from metastases both *in vivo * and *ex-vivo.*

In the dorsal and ventral whole body BLI images, the 66c14 luc2 and 4 T1.2 luc3 cells were visible in the primary tumors and numerous secondary metastatic regions *in vivo* using a BLI intensity imaging scale on the order of 10^6^ photons/sec/cm^2^ (highlighted with a yellow box in Figure 5A). On the other hand, in whole body BLI images of mice injected with the commercially available 4 T1 luc2 cells only primary tumors were visible. The high bioluminescence produced by the 4 T1 luc2 primary tumor was imaged using a scale on the order of 10^8^ photons/sec/cm^2^ (highlighted with a red box in Figure 5A), and likely prohibited secondary sites of metastases to be visualized.

*Ex vivo* analyses were also performed in order to identify specific tissues where metastases formed. BLI revealed 66c14 luc2 *ex vivo* metastases primarily to lung, although metastases to liver and spine were detectable, while 4 T1.2 luc3 cells were highly metastatic to lung, liver and spine (Figure 5B, Figure 3). Mice injected with the commercially available 4 T1 luc2 cells showed weak *ex vivo* BLI intensities from metastases in the lung, liver, and spleen, and no spine metastases were measured using the same scale as with the 66c14 luc2 and 4 T1.2 luc3 cells (Figure 5B, yellow box; Figure 3).

**Figure 6:**
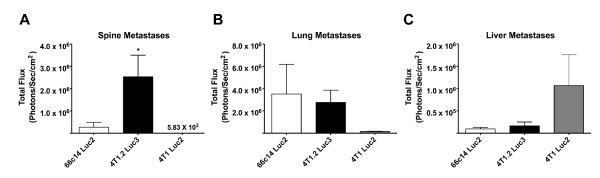
**Quantification of BLI intensity from *ex-vivo *66c14 luc2, 4 T1.2 luc3, and 4 T1 luc2 metastatic organs.** Bioluminescence intensities were quantified *ex vivo *in **(A)** spine, **(B)** lung, and **(C)** liver of mice orthotopically injected with 66c14 luc2 (white bars), 4 T1.2 luc3 cells (black bars), or 4 T1 luc2 (grey bars), 32 days (4 T1.2 luc3 and 66c14 luc2) or 28 days (4 T1 luc2) or after tumor cell injection. Spines from mice injected with 4 T1.2 luc3 cells showed significantly higher BLI intensity as compared to both 66c14 luc2 and 4 T1 luc2 injected mice. Data expressed as photons/sec/cm^2^ (mean ± SEM; *n = 3-4, * *p < 0.05, unpaired t-test).

Quantification of the BLI intensities from spines, lung, and liver *ex vivo* from mice injected with 66c14 luc2, 4 T1.2 luc3, and 4 T1 luc2 cells were performed. Results showed that spines excised from the 4 T1.2 luc3 group had BLI intensities 10-fold higher than the 66c14 luc2 group and three orders of magnitude (10^3^) higher than that of the 4 T1 luc2 injected group (Figure 6A). Lungs from mice injected with either 66c14 luc2 or 4 T1.2 luc3 cells had a mean BLI intensity an order of magnitude higher than the 4 T1 luc2 (Figure 6B). Interestingly, one of the mice injected with 66c14 luc2 cells also showed a high average BLI intensity from multiple metastases in the liver, never previously reported in the parental 66c14 cell line (Figure 6C). Overall, the metastatic burden, which accounts for the average number and size of metastases, detected *in vivo* and *ex vivo* was highest for 4 T1.2 luc3 cells. In terms of their ability to be tracked in the primary tumor and secondary metastatic sites *in vivo* and *ex vivo*, both 4 T1.2 luc3 and 66c14 luc2 cells showed higher BLI intensities than 4 T1 luc2 cells.

### Micro-computed tomography (Micro-CT) analysis of 4 T1.2 luc3 cells

**Figure 7:**
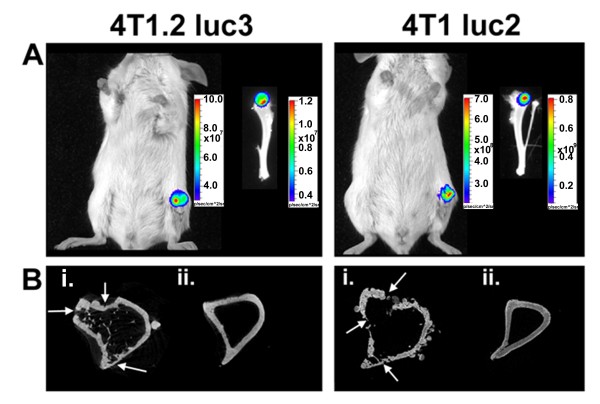
**4T1.2 luc3 cells are osteolytic *in vivo. *(A) ** 1 × 10^4^ 4 T1.2 luc3 (left) or 4 T1 luc2 cells (right) were injected into the proximal end of the left tibia of a 6-week old female Balb/c mice and visualized after i.p. injection of 150 mg/kg D-Luciferin using an IVIS Spectrum BLI system (day 18 after tumor cell injection). Whole body ventral view shows luciferase activity from tumor cells in the left tibia, validated with *ex-vivo *imaging of the tibia. **(B)** micro-CT images of cross sectional slices (4000 × 4000 pixels) **(i)** at the proximal and **(ii)** distal ends of the tibia reveal extensive osteolytic bone degradation (osteolytic damage highlighted with white arrows) near the injection site. Micro-CT images scanned using a Microphotonics Skyscan 1172 CT at a 6μm resolution and reconstructed using ImageVis3D imaging software.

To identify whether 4 T1.2 luc3 cells are osteolytic by comparison to the commercially available 4 T1 luc2 cells, each of the cell lines were injected into the tibia of 6-week old female Balb/c mice and analyzed for bone integrity using micro-CT. Whole body and *ex vivo* BLI analysis of mice injected with 4 T1.2 luc3 and 4 T1 luc2 cells verified the presence of tumor cells 18 days after injection into the tibia (Figure 7A). Micro-CT analysis showed significant osteolytic activity of both cell lines near the injection site at the proximal tibia (Figure 7B-i) and normal bone at the distal tibia (Figure 7B-ii). 66c14 luc2 cells were not analyzed for osteolytic potential in this study. Overall, these results suggest that 4 T1.2 luc3 cells are a useful model for studying osteolytic bone metastasis originating from a primary breast tumor.

## Discussion

This study presents two new luciferase-expressing mouse cell lines (66c14 luc2 and 4 T1.2 luc3 cells) useful for the *in vivo* study of breast cancer metastasis to bone. Using BLI, these cell lines demonstrate greater bone metastatic potential in a syngeneic Balb/c mouse model than the commercially available 4 T1 luc2 cell line (Caliper Life Sciences, Hopkinton, MA). The 4 T1.2 luc3 cell line is metastatic to the lung, liver, and bone in 100% of the mice tested, and the 66c14 luc2 cell line is highly metastatic to lung and less metastatic to bone based on BLI intensities. Each of the two cell lines showed *in vitro* growth rates (data not shown) and *in vivo* tumor sizes similar to the parental 4 T1.2 and 66c14 cell lines.

Lung and bone are the predominant destinations of metastatic 4 T1.2 cells [10,11]. However, here we report detectable metastases in the liver of mice injected with the 4 T1.2 luc3 cell line as well as the 66c14 luc2 cell line, a site of metastases never previously identified with the parental 4 T1.2 or 66c14 cells although demonstrated with the 4 T1 cells [10]. It is possible that these cells have increased metastatic potential due to incorporation of the luciferase gene and/or due to the transfection and clone selection process. This increased metastatic potential displayed by the luciferase-expressing cell lines is further supported by our ability to detect low levels of bioluminescence from the spines of mice treated with the 66c14 luc2 cells, when previous studies have not shown detectable metastases to bone using the parental 66c14 cell line [10]. However, it is important to note that the bioluminescence intensity from metastatic 4 T1.2 luc3 cells in the spine was nearly ten-fold greater than that of the 66c14 luc2 cells and three orders of magnitude greater (10^3^) that that of the 4 T1 luc2 cells, although the number of metastases detected was similar for all three cell lines (data not shown). This could be partially explained by differences in tumor growth rate and size at the endpoint of the experiment of mice injected with the 4 T1.2 luc3 cells compared to the 66c14 luc2 or 4 T1 luc2 cells (data not shown). In general, a slower progression of the primary tumor has been correlated with increased metastasis in mouse models [5].

Orthotopically injected 66c14 luc2 or 4 T1.2 luc3 cells outperformed 4 T1 luc2 cells in whole-body BLI for the detection of metastases. Bioluminescence was detected in the thoracic and abdominal regions of whole mouse images suggesting that metastasis of 66c14 luc2 and 4 T1.2 luc3 cells had occurred. This was verified by the presence of metastases in the lungs, spine, and liver *ex vivo*. There was no bioluminescence detected in these regions of the mice injected with the 4 T1 luc2 cells using IVIS instrument settings appropriate for visualizing the primary tumor. Although there were verified metastases detected in the lung, spine, and liver by *ex vivo* imaging from mice injected with the 4 T1 luc2 cells, the intensities were several orders of magnitude lower than the primary tumor. It is important to note that the much higher luminescence of the 4 T1 luc2 primary tumor makes bioluminescence visualization and quantification of secondary sites of metastasis in the whole mouse difficult.

To establish an *in vivo* model system where the number of metastatic sites was increased by bypassing initial steps of the metastatic cascade, intra-cardiac injections were performed. Injection of 4 T1.2 luc3 cells into the left ventricle resulted in cell migration to and colonization in lung, liver, spine, brain, spleen, and kidneys (data not shown). The same metastatic sites were seen with 4 T1 luc2 cells and a similar pattern was seen with 66c14 cells, with the exception of spleen and brain (data not shown). therefore 4 T1.2 luc3 cells are particularly useful for investigations of mammary tumor metastatic sites including brain and kidney if administered via intra-cardiac injection.

Intra-tibial injections of 4 T1.2 luc3 and 4 T1 luc2 cells were performed to evaluate the osteolytic nature of these cells. Parental 4 T1 cells have been extensively characterized *in vivo* for their osteolytic activity in bone [15-17], while no previous publications address either parental 4 T1.2 or 4 T1 luc2 cells. Our results demonstrated that both 4 T1.2 luc3 and 4 T1 luc2 cells have similar osteolytic activity *in vivo* (Figure 7). Therefore, these cells are useful in BLI and 3-D bone morphology studies performed in tandem investigating not only metastasis, but also the osteolytic capacity of mammary tumor cells.

## Conclusions

The purpose of this study was to incorporate the luciferase gene into previously characterized mouse mammary tumor cell lines with discrete bone metastatic profiles in order to track metastases *in vivo* using BLI. We show that the highly metastatic 4 T1.2 luc3 cell line, when injected orthotopically, closely models the sites of metastases seen in human patients including lung, liver, and bone. These cells show osteolytic activity *in vivo* and the ability to proliferate in additional organ sites such as brain, spleen, and kidneys. We also present results demonstrating a phenotypically similar 66c14 luc2 cell line that shows similar metastases in the lung as the 4 T1.2 luc3 cell line along with previously unreported metastases in bone. These imageable cells lines are powerful tools for identifying mechanisms important for breast cancer metastasis to bone *in vivo*.

## Methods

### Cells lines and cell culturing

66c14 and 4 T1.2 cells were cultured and maintained in α-MEM supplemented with 10% fetal bovine serum, 1 mM Penicillin/Streptomycin, and 1 mM sodium pyruvate at 37°C in 5% CO_2_ and 95% humidity. 4 T1 luc2 cells were purchased from Caliper Life Sciences (Hopkinton, MA) and grown according to manufacturer’s instructions.

### Stable transfections of cells containing the pGL4 vector

Approximately 2.5 × 10^5^ 66c14 and 4 T1.2 cells were plated in 6-well culture dishes and transfected with 5.0 μg of pGL4.13[*luc2*/SV40]vector (Promega, Madison, WI) using Lipofectamine LTX (Invitrogen, Carlsbad, CA) per manufacturer’s instructions. The cells were trypsinized (0.25% trypsin) 48 hours after transfection, diluted, and plated into 96-well plates. Two weeks after plating, 100 μL media containing D-luciferin (Caliper Life Sciences, Hopkinton, MA) at a final concentration of 150 μg/mL was added to the 96-well plates. Visualization of bioluminescence was performed using the Kodak Image Station 4000R (Eastman Kodak Company, Rochester, NY) with a 5-minute exposure. Bioluminescent cells were serially diluted into 96-well plates and cells were grown approximately 2 weeks and assayed for bioluminescence. The colonies with the highest levels of bioluminescence were diluted to 1 cell/100 μL and plated in 96-well plates, grown, and assayed again for bioluminescence. To ensure a clonal population, the colonies with the highest level of bioluminescence were again selected, and the above process was repeated an additional time. Finally, the colonies with the highest level of bioluminescence were selected and expanded to obtain clonal populations of 66c14 luc (66c14 luc1 to 66c14 luc5) and 4 T1.2 luc (4 T1.2 luc1 to 4 T1.2 luc5) cells expressing the luciferase gene.

### *In vitro* bioluminescence assay to estimate cell line bioluminescence intensities

Approximately 1 × 10^5^ 66c14 luc and 4 T1.2 luc cells containing stably expressing the pGL4 vector, as well as control HeLa luc cells (Caliper Life Sciences) were plated in a 24-well culture dish and incubated overnight. Bioluminescent intensity was measured as described in the previous section. Using ImageJ software (National Institutes of Health), each captured image was inverted and total pixel density was calculated. HeLa luc cells, producing approximately 350 photons/sec/cell, were used as a reference point to estimate the photons/sec/cell produced by each of the new 66c14 luc and 4 T1.2 luc cell lines.

### Injection of cells for measuring *in vivo* bioluminescence imaging (BLI) and tumor progression

All animal studies were conducted in accordance with the animal component of research protocol (ACORP) #JOR0010 protocol approved by the Institutional Animal Care and Use Committee (IACUC) at the Boise VA Medical Center, Boise, ID. 1 × 10^5^ cells suspended in 10 μL of phosphate buffered saline (PBS) were injected into the 4^th^ mammary fat pad of 7-week old female Balb/c mice (n = 3/group). Ten different groups of cells were injected including parental (control) 66c14, 66c14 luc2, 66c14 luc3, 66c14 luc4, 66c14 luc5, parental (control) 4 T1.2, 4 T1.2 luc1, 4 T1.2 luc2, 4 T1.2 luc3, and 4 T1.2 luc4 cells. Tumor size was measured with calipers bi-weekly once tumors became palpable (after approximately 2 weeks). BLI of live animals was initiated at 18 days after cell line injection and performed weekly. Briefly, mice were injected i.p. with 150 mg/kg of D-luciferin (Caliper Life Sciences) in PBS, anesthetized with 2.5% isoflurane, and imaged. Mice were imaged using a charge-coupled device camera–based bioluminescence imaging system (IVIS Spectrum, Caliper Life Sciences; exposure time 1–300 sec, binning 4/8/16, field of view 23 cm, f/stop 1, emission filter open). Signal was measured and recorded as total flux (photons/sec). Corresponding grayscale photographs and color luciferase images were automatically superimposed and analyzed with Living Image software (Xenogen Biosciences Corporation, Cranbury, NJ).

Mice utilized for metastasis investigations were treated exactly as above with the following modifications. Three mice per cell line were orthotopically injected with 66c14 luc2, 4 T1.2 luc3, or 4 T1 luc2 cells. Whole animals were imaged 4–5 weeks following tumor cell injection and immediately injected with a second dose of 150 mg/kg of D-luciferin. Ten minutes following this injection the mice were euthanized with isoflurane gas followed by cervical dislocation, and the lungs, spine, spleen, and liver were dissected and imaged *ex vivo* using the bioluminescence imaging system (exposure time 1–300 sec, binning of 4/8/16, field of view 23 cm, f/stop of 1, emission filter open).

### Micro-computed tomography (Micro-CT)

In order to verify the osteolytic potential of the 4 T1.2 luc3 cells, 1 × 10^4^ cells in 100 μL of PBS were injected into the left tibia of a 6-week old female Balb/c mouse. *In vivo* bioluminescence was determined as stated above. Immediately following *ex-vivo* imaging of the tibia, it was placed in 10% formalin overnight and stored in 70% ethanol for analysis. Micro-CT analysis was conducted using a Microphotonics Skyscan 1172. A 6.6 μM resolution was used for image collection of 2D scans (4000 × 4000 pixels) and ImageVis3D imaging software (http://www.imagevis3d.org) for 3D reconstructions.

## Competing interests

The authors declare that they have no competing interests.

## Authors’ contributions

CS was responsible for the *in vitro* characterization of the luciferase-expressing 4 T1.2 and 66c14 cell lines as well as the initial *in vivo* tumor progression studies. KT. was responsible for the transfection and selection of the luciferase-expressing 4 T1.2 and 66c14 cell lines. CB was responsible for the 4 T1.2 luc3 and 66c14 luc2 *in vivo* and *ex vivo* metastasis data along with the majority of all writing and figure compilations. JM was responsible for the 4 T1 luc2 *in vivo* metastasis study. CJ was the primary investigator responsible for directing all the work conducted in this manuscript. All authors read and approved the final manuscript.

## Authors’ information

Caleb Sutherland is currently a PhD graduate student in the Department of Cancer Biology at the University of Arizona in Tucson, AZ. Caleb conducted his research contributing to this manuscript while a McNair Scholar working in the laboratory for Dr. Cheryl Jorcyk at Boise State University. Ken Tawara, M.S. received his Master’s degree in the laboratory of Dr. Cheryl Jorcyk in March of 2011 and performed the research pertaining to this manuscript while conducting his graduate studies. Dr. Celeste Bolin and Dr. Jim Moselhy are post-doctoral fellows in the laboratory of Dr. Cheryl Jorcyk. Dr. Cheryl Jorcyk is a full professor at Boise State University in the Department of Biological Sciences and collaborates with Dr. Robin Anderson who is head of the Metastasis Research Laboratory at the Peter MacCallum Cancer Centre in Melbourne, Australia.
